# Mesenchymal stem cells significantly improved treatment effects of Linezolid on severe pneumonia in a rabbit model

**DOI:** 10.1042/BSR20182455

**Published:** 2019-09-16

**Authors:** Dexiao Kong, Xia Liu, Xiaomei Li, Jianting Hu, Xiaoyan Li, Juan Xiao, Yibo Dai, Mingming He, Xiaoli Liu, Yang Jiang, Ruodi Cui, Lihong Zhang, Juandong Wang, Ai Li, Fang Wang, Yuan Zhang, Juan Xiao, Wei Wang, Chengyun Zheng

**Affiliations:** 1 Department of hematology of the Second Hospital, Institute of Biotherapy for Hematological Malignancies, Shandong University-Karolinska Institute Collaborative Laboratory for Stem Cell Research, Shandong University, Jinan, Shandong Province, China; 2 Department of Hematology, Zhaoyuan Sorting-Yingcheng Hospital, Second Hospital of Shandong University, Yantai, Shandong Province, China; 3 Department of Respiratory Intervention, Qilu Children’s Hospital of Shandong University, Jinan, Shandong Province, China; 4 Cancer Center, The Second Hospital of Shandong University, Jinan, Shandong Province, China; 5 Shandong Pharmaceutical Academy, Shandong Provincial Key Laboratory of Chemical Drugs, Jinan, Shandong Province, China; 6 Department of Radiology, Qilu Children’s Hospital of Shandong University, Jinan, Shandong Province, China; 7 Department of Pathology, Qilu Children’s Hospital of Shandong University, Jinan, Shandong Province, China; 8 Clinical Laboratory, The Second Hospital of Shandong University, Jinan, Shandong Province, China; 9 Center of Evidence-Based Medicine, The Second Hospital of Shandong University, Jinan, Shandong Province, China; 10 Department of Respiratory Medicine, The Second Hospital of Shandong University, Jinan, Shandong Province, China

**Keywords:** linezolid, Mesenchymal stem cells, MRSA, rabbit model, Staphylococcus aureus, synergistic effect

## Abstract

The present study aimed to investigate whether co-administration of mesenchymal stromal cells (MSC) and linezolid (LZD) into a rabbit model of methicillin-resistant *Staphylococcus aureus* (MRSA)-infected pneumonia would bring a synergistic therapeutic effect. Human umbilical cord-derived MSCs (hUMSCs) were isolated and characterized. A rabbit model of pneumonia was constructed by delivering 1 × 10^10^ CFU MRSA via a bronchoscope into the basal segment of lower lobe of right lung. Through analyzing vital sign, pulmonary auscultation, SpO_2_, chest imaging, bronchoscopic manifestations, pathology, neutrophil percentage, and inflammatory factors, we verified that a rabbit model of MRSA-induced pneumonia was successfully constructed. Individual treatment with LZD (50 mg/kg for two times/day) resulted in improvement of body weight, chest imaging, bronchoscopic manifestations, histological parameters, and IL-10 concentration in plasma (*P<*0.01), decreasing pulmonary auscultation, and reduction of IL-8, IL-6, CRP, and TNF-α concentrations in plasma (*P<*0.01) compared with the pneumonia model group at 48 and 168 h. Compared with LZD group, co-administration of hUMSCs (1 × 10^6^/kg for two times at 6 and 72 h after MRSA instillation) and LZD further increased the body weight (*P<*0.05). The changes we observed from chest imaging, bronchoscopic manifestations and pathology revealed that co-administration of hUMSCs and LZD reduced lung inflammation more significantly than that of LZD group. The plasma levels of IL-8, IL-6, CRP, and TNF-α in combined group decreased dramatically compared with the LZD group (*P<*0.05). In conclusion, hUMSCs administration significantly improved therapeutic effects of LZD on pneumonia resulted from MRSA infection in a rabbit model.

## Introduction

Methicillin-resistant *Staphylococcus aureus* (MRSA), isolated frequently from both community and hospital settings, is one of the major pathogenic microorganisms for nosocomial infections that lead to considerable morbidity and mortality [1,2]. Although linezolid (LZD) was recommended as a standard choice for the treatment of MRSA nosocomial pneumonia [3], the ratio of morbidity and mortality associated with MRSA infections is still high [4]. To date, the best treatment for this potentially life-threatening infection has not been definitely defined. Given the serious clinical and public health problem, novel as well as effective therapies are urgently needed.

Currently, more and more researches demonstrated that the severe pneumonia involves in the imbalance between pathogen and immune system of the host [5]. The complicated inflammatory responses accompanied by release of multiple inflammatory cytokines, named cytokines storm, causing multiorgan failure, collapse of the circulatory system, and death [6]. Thus, the interest in immunotherapy alternatives and its potential role in treating MRSA infections is arising.

Mesenchymal stem cells (MSCs) are multipotent stem cells classically isolated from the bone marrow. The potent immunomodulatory properties of MSCs [7,8] including the inhibition of pro-inflammatory processes in response to endotoxin and bleomycin, profound immunosuppression by inhibiting T-cell responses to polyclonal stimuli. It has been proved that MSCs can reduce the infiltration and aggregation of the neutrophils, monocytes-macrophages in the lung. Additionally, it can decrease the level of inflammatory cytokines such as tumor necrosis factor (TNF)-α, TNF-β, Interleukin (IL)-1, and IL-6 [9], and increase the expression of anti-inflammatory cytokines such as IL-10 and cyclooxygenase-2 [10]. In the acute lung injury (ALI) researches, it has been confirmed that MSCs can reduce the expression of IL-1, IL-6, and TNF-α in the ALI model induced by bleomycin [11]. Chen et al. [12] also found the down-regulation of TNF and the up-regulation of IL-10 in the ALI model induced by phosgene after MSCs transfusion.

Based on these studies, we wondered whether MSCs could assist in bringing disrupted inflammatory responses back into balance, thus preventing MRSA-induced pneumonia from progressing to severe pneumonia, multiorgan failure, and even death. Thereby, we investigated the synergistic effect of MSCs combined with LZD in MRSA-induced severe pneumonia in a rabbit model.

## Material and methods

### Animals

About 54 healthy young male New Zealand white outbred rabbits (3–6 months old, median 3.17 kg weight [2.9–3.38 kg]), which were purchased from Xilingjiao Breeding Center of Jinan (production license number: SCXK [Lu] 20150001), were used in the present study. Rabbits were housed in individual cages, with controlled temperature and humidity, and a 12-h light/dark cycle. They were fed with a standard chow and water *ad libitum*.

### Bacterial strains and culture conditions

MRSA strain, ATCC 33591, was a lyophilized strain that purchased from American Type Culture Collection (ATCC, LOT 2300122). MRSA was dissolved and grown in the blood plate. Then a single clone was cultured and amplified in sterile liquid Luria-Bertani medium at 37°C with shaking 225 rpm. Then MRSA was maintained in 0.9% sterile saline containing 10% glycerol to a concentration of 1–5 × 10^10^ colony forming units (CFU) per milliliter at −80°C. The concentration of the bacteria was verified by tittering the frozen stocks in triplicate on three separate occasions. For inoculation of rabbits, the corresponding amounts of bacteria were injected to basal segment of rabbit right lower lobe from the bronchial biopsy hole.

### Allogeneic umbilical cord-derived human MSC isolation, purification, and identification

hUMSCs were separated, cultured, and identified as we described previously [13]. Briefly, umbilical cords were obtained from healthy puerpera. The donors had no genetic family or cancer history. The donor’s sera were assessed to exclude HBV, HCV, HIV, EBV, CMV, and syphilis infection. The preparation of hUMSCs was performed under the standard of the Good Manufacturing Practice laboratory. The isolated hUMSCs were cultured in 90% DMEM (Gibco; Thermo Fisher Scientific, Inc.) supplemented with FBS (10%, Gibco; Thermo Fisher Scientific Inc.; cat no. 10100147), penicillin (100 U/ml), streptomycin (100 μg/ml), and cytokine mixture (secret recipe) at 37°C with 5% CO_2_. After 3 days cultivation, non-adherent cells were removed and culture medium was replaced. To determine the phenotype of P4 hUMSCs, the expression of surface markers CD90, CD73, CD105, CD166, CD14, CD19, CD34, CD45, and HLA-DR was analyzed by BD FACS calibur (BD Biosciences, Franklin Lakes, NJ. U.S.A.). As multipotent differentiation potential is one of the criteria to define MSC [14], we then performed differentiation assays using Differentiation Kits (Invitrogen Life Technologies, Carlsbad, CA, U.S.A.) to assess whether the purified MSC could differentiate to osteogenic, chondrogenic, and adipogenic lineages *in vitro* under standard differentiating conditions.

The fifth generation was utilized in the *in vivo* experiments. When cells grow to 80% confluence, they were digested by trypsin and washed by NS for three times. Single cell suspension was then prepared using NS to a concentration of 1.5 × 10^7^/ml and used for intravenous injection.

### Rabbit anesthesia and sedation

For bronchoscopy, animals were sedated with 3 mg/kg midazolam (Nhwa Pharmaceutical Corporation Ltd., Jiangsu, China) by intravenous injection. After rabbits were narcotized, 0.5 ml of 2% lidocaine was dropped into nasal cavity for local anesthesia before operation. The anesthesia process is performed by a professional anesthesiologist and the anesthetic effect was assessed. Anesthesia observation indicators include smooth breathing, muscle relaxation, no pain response, and miosis. The 3–5 l/min oxygen flow was used to maintain percutaneous oxygen saturation (SpO_2_) more than 90% with face mask, and heart rate, breathing, pulse, and (SpO_2_) were intraoperative monitored using MINDRAY BeneView T8 monitor (Shenzhen, China). For necropsy, animals were euthanized with 2% pentobarbital sodium 70 mg/kg by intravenous injection.

### Construction of rabbit pneumonia model

Briefly, 18 healthy young male New Zealand white outbred rabbits were formulated into two cohorts of nine animals and randomly assigned to receive normal saline (NS) or 1 × 10^10^ CFU MRSA. A bronchoscope (EVIS LUCERA BF-XP260F, Olympus, Japan) was used for infections by passing into the trachea of anesthetized rabits and positioned into the first bronchiole of the lateral bronchiole system that forms the basal segment of lower lobe of right lung. After instillation of 1 × 10^10^ CFU MRSA strain in 1 ml inoculum, the rabbits were quickly kept in sitting posture and flapped the right lower lung to ensure the bacteria into the distal bronchiole. At the same time, rabbits were given aerosol inhalation with 1.25 mg salbutamol and 0.5 mg budesonide to reduce airway spasm and inflammatory edema. Rabbits were kept under standard conditions and observed hourly by trained veterinary staff. Three rabbits randomly picked animals from each treatment group were killed at 48 and 168 h. Lungs were removed aseptically from euthanized rabbits and cut into <0.5 cm pieces. The preinfection and perimortem tests contained vital sign measurement, pulmonary auscultation, SpO_2_, CT, bronchoscopic manifestations, pathology, neutrophils, and inflammatory factors, such as C-reactive protein (CRP), procalcitonin (PCT), IL-8, IL-6, TNF-α, and IL-10.

### hUMSCs and LZD treatment on rabbit pneumonia

We observed the synergistic effect of co-administering hUMSCs and LZD into a rabbit model on MRSA-infected pneumonia. Rabbits were radomly divided into four groups: (1) combined group: co-administration of hUMSCs and LZD to therapy MRSA induced pneumonia; (2) LZD group: intravenous infusion of LZD to therapy MRSA induced pneumonia; (3) model group: 1 × 10^10^ CFU MRSA strains were instilled into the basal segment of lower lobe of right lung to construct rabbit pneumonia; and (4) control group: NS only was instilled. There were nine rabbits in each group. hUMSCs (1 × 10^6^/kg) were administered by intravenous injection through ear vein at 6 and 72 h after MRSA instillation. LZD (Fresenius KabiNorge As, Halden, Norway) was administered intravenously for two times/day (50 mg/kg) from 6 h after MRSA instillation to 7 d through ear vein. The examinations of preinfection and separate time points after postinoculation were conducted, including vital sign measurement, pulmonary auscultation, SpO_2_, chest imaging, bronchoscopic manifestations, pathology, neutrophils, and inflammatory factors (CRP, PCT, IL-8, IL-6, IL-10, and TNF-α).

### Chest imaging

Chest CT scan was conducted with a 128-slice spiral scanner (Somatom definition AS, Siemens Medical Systems, Forchheim, Germany) before and at 0, 48, and 168 h after inoculation. Intravenous injection of Midazolam (3 mg/kg) before CT scan to calm down the rabbits in a prone position during scanning. The scans were observed at the standard lung window (level –600 HU, width 1200 HU) and mediastinal window (level 50 HU, width 350 HU).

### Histopathology examination

The whole lung and each pulmonary lobe were collected at the time of death or euthanasia before and after modeling for 48 and 168 h, respectively. Then lung tissues were fixed with 10% buffered formalin. Hematoxylin and eosin-staining was performed according to standard methods.

### Gross and microscopic pathological analyses

Tissues collected at necropsy were analyzed by pathologists and veterinarians. A lung gross pathology score was computed according to five criteria as described previously [15]. Hematoxylin and eosin-stained sections were observed using a BX5 microscope and photographed with a DP70 camera (Olympus Imaging America). Lung histopathology was evaluated independently by two pathologists. A lung microscopic pathology score was computed for each animal based on four criteria as previous description [15].

### Measurements of white blood cell and neutrophil

Blood with EDTA anticoagulant was collected before and after modeling for 6, 24, 48, 72, 120, and 168 h, respectively. Then routine blood test was conducted using the fully automatic hematology analyzer (SYSMEX XT-2000iv, Japan) to explore the change of white blood cells and neutrophils. The neutrophil percentage was calculated as follows: (the number of neutrophils/the total number of white blood cells) × 100%.

### Measurements of cytokines concentrations in plasma

Blood with heparin anticoagulant was collected before and after modeling for 6, 24, 48, 72, 120, and 168 h, respectively. Plasma concentrations of pro-inflammatory cytokines (CRP, PCT, IL-8, IL-6, and TNF-α) and anti-inflammatory cytokine IL-10 were determined by the ELISA using Rabbit-Specific Kits (Cusabio biotech corporation, China) following the manufacturer’s instructions. In brief, 100 μl of experiment samples or standard samples were added into each well of the reaction plate and incubating for 2 h at 37°C. Then the original solution of wells was discarded and 100 μl biotin-labeled antibody working solution was added to each wells for 1 h incubation at 37°C. Following that, the plates were washed for three times and added with horseradish peroxidase-labeled avidin for 15 min incubation at 37°C in the dark. Then the reaction was terminated by stop buffer. The absorbance at 450 nm was detected using a SpectraMax i3X Multi-mode detection platform (Molecular Devices, Inc., U.S.A.). The standard curve was plotted using the OD value and the concentrations of the standard samples. The concentrations of CRP, PCT, IL-8, IL-6, TNF-α, and IL-10 were calculated based on the standard curve.

### Statistical analysis

All experimental data were shown as mean ± S.D. The effect of each treatment group was analyzed by variance analysis of repeated measurement data. ANOVA was used to compare the difference amongst the groups at different time points. Statistical comparisons were performed using LSD test at separate time points for each strain management group. *P<*0.05 was considered to be statistically significant.

## Results

### Establishment of rabbit pneumonia model and clinical assessment of MRSA-infected rabbits

**Figure 1:**
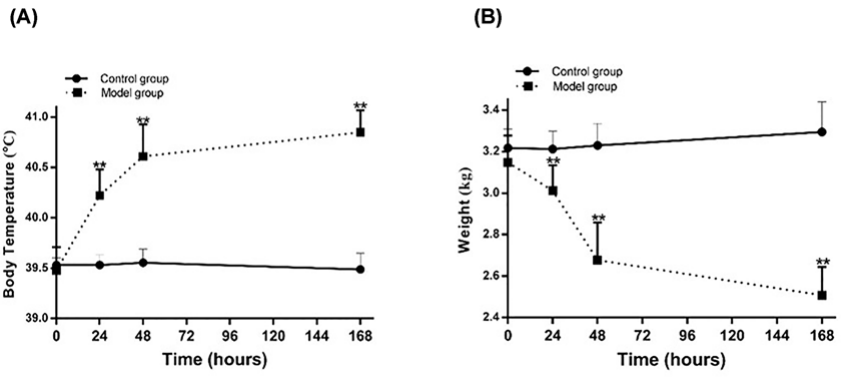
Change of temperature and weight in the pneumonia model of rabbit (**A**) Temperature change after MRSA inoculation within 168 h. (**B**) Weight change after MRSA inoculation within 168h. *P<0.05, **P<0.01 compared with control group.

In the early stage of our study, we investigated the degree of pneumonia caused by different concentrations of MRSA (conrol group [normal saline]; low dose group [1 × 10^8^ CFU], middle dose group [1 × 10^9^ CFU], high dose group [1 × 10^10^ CFU], and extremely high dose group [5 × 10^10^ CFU]). We found that endotracheal directed instillation of 1 ×10^8^ and 1 × 10^9^ CFU MRSA in outbred rabbits through bronchoscope resulted in no mortality. Whereas treatment with 1 × 10^10^, and 5 × 10^10^ CFU MRSA caused 30–100% mortality within 2 weeks. As 5 × 10^10^ CFU MRSA caused 100% mortality within 2 weeks; hence, this concentration is not suitable for rabbit pneumonia model construction. Except for the control group, the other three groups (low/middle/high dose groups) had different degrees symptoms of the lower respiratory tract infection, such as cough, nasal yellow secretions, shortness of breath, wet rales of right lung bottom, decreasing food intake and vitality, weight loss, decreased SpO_2_, and fever (data not shown). Through vital sign measurement, pulmonary auscultation, SpO_2_, chest imaging, bronchoscopic manifestations, pathology, neutrophils, and inflammatory factors analyses, we demonstrated that rabbits in low, middle, and high dose groups could form pneumonia at 48 h after MRSA instillation, and the severity of pneumonia is positively correlated with the dose of MRSA applied (data not shown). In our present study, we aimed to construct a model of severe pneumonia and observe the synergistic therapeutic effect of LZD and hUMSCs. Therefore, high dose group (1 × 10^10^ CFU MRSA) was selected as the model group in the present study. Our results showed that the body temperature of the rabbits in model group began to increase gradually from 24 h and peaked at 168 h (*P<*0.01, Figure 1A). The body weight of the rabbits in model group decreased significantly and reached the lowest point at 168 h (*P<*0.01, Figure 1B). All MRSA-infected rabbits developed a significant lower respiratory tract infection; however, the control rabbits remained their baseline level of health (Table 1). Except showing signs of toxic shock during the 48 h postinoculation observation period, the high dose MRSA-infected animals exhibited other non-specific signs of infection such as increased daytime somnolence, decreased food intake, loss of weight, and decreased the excretion of urine and feces. They also had a frequent cough and more yellow nasal secretions (8/9, Table 1). At the same time, we could hear coarse breath sounds and right lung basilar wet rales, more prominent over the right lower basal segment (inoculation site) and lower lung lobe during expiration, consistent with airway obstruction. Importantly, the examinations at 48 h revealed markedly wet rales of lung in all MRSA-infected rabbits (Table 1). Compared with the sham-inoculated control animals, the MRSA-infected animals had reduced hemoglobin oxygen saturation, suggesting a defect in alveolar gas exchange (Table 1).

**Table 1:** Clinical-pathological data of MRSA-infected rabbit pneumonia and treatment effect of UMSC combined with LZD

Treatment	Cough		Positive pulmonary signs		Loss of weight (>10%)		Increased temperature (>10%)		Decreased oxygen saturation (<96%)	
	48 hpi	168 hpi	48 hpi	168 hpi	48 hpi	168 hpi	48 hpi	168 hpi	48 hpi	168 hpi
**Control group**	0/9	0/6	0/9	0/6	0/9	0/6	0/9	0/6	0/9	0/6
**Model group**	8/9	6/6	7/9	6/6	9/9	6/6	8/9	5/6	9/9	6/6
**LZD group**	4/9	1/6	6/9	0/6	8/9	1/6	5/9	1/6	5/9	0/6
**Combined group**	4/9	0/6	4/9	1/6	7/9	0/6	3/9	0/6	4/9	0/6

The overall clinical-pathological characteristics of the rabbits were recorded as the number of events/total number of animals per group. Significant alterations were defined as: markedly wheezing breath sounds or marked wet rales being heard by lung auscultation; oxygen saturation being measured at less than 96% by pulse oximetry; loss of weight more than 10%; increased temperature more than 10% (Olsen RJ, Ashraf M, Gonulal VE, Ayeras AA, Cantu C, Shea PR, Carroll RK, Humbird T, Greaver JL, Swain JL. *Lower respiratory tract infection in cynomolgus macaques [Macaca fascicularis] infected with group A Streptococcus.* Microb. Pathog., 2010. **49**(6): p. 336-347].

Abbreviations: hpi, hours post inoculation; UMSC, Umbilical cord derived mesenchymal stem cell.

**Figure 2:**
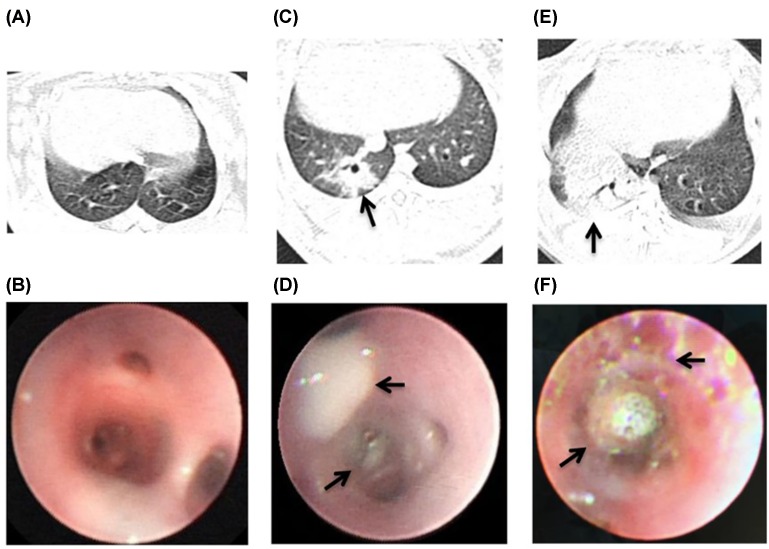
The performance of bronchoscopy and CT in the pneumonia model of rabbit (**A**) The chest CT appearance of normal rabbit. (**B**) The right lung lower basal segment of NS sham-infected rabbits had a normal appearance. (**C**) The right lower lung basal segment of MRSA-infected rabbits has lamellar lung consolidation, centrilobular nodules at 48 h postinoculation (arrow). (**D**) The bronchioles of MRSA-infected rabbits had prominent tracheal constriction, bronchial mucosal purulent lesions at 48 h postinoculation (arrow). (**E**) CT showed the consolidation of right lower lung of model group at 168 h postinoculation (arrow). (**F**) The purulent secretions blocked the lumen and bronchial erythema, erosions, mucosal congestion and edema in the model group at 168 h postinoculation (arrow).

Moreover, compared with the chest CT of the control group (Figure 2A), the right lower lung basal segment of the model group has lamellar lung consolidation, centrilobular nodules 48 h postinoculation (Figure 2C). From bronchoscopy examination, we observed that compared with the control group (Figure 2B), all MRSA-infected rabbits developed bronchial erythema, mucosal congestion and edema, and superficial bronchial erosions with purulent lesions at 48h postinoculated with 1 × 10^10^ CFU MRSA into right lung lower basal segment (Figure 2D). With the extension of modeling time, the right lower pneumonia gradually aggravated and increased further at 168 h (Figure 2E). And the performance of bronchoscopy gradually aggravated. The purulent secretions blocked the lumen, and further aggravated bronchial mucosal congestion and edema in the model group at 168 h (Figure 2F).

### Gross pathology of the MRSA-infected rabbit lung

**Figure 3:**
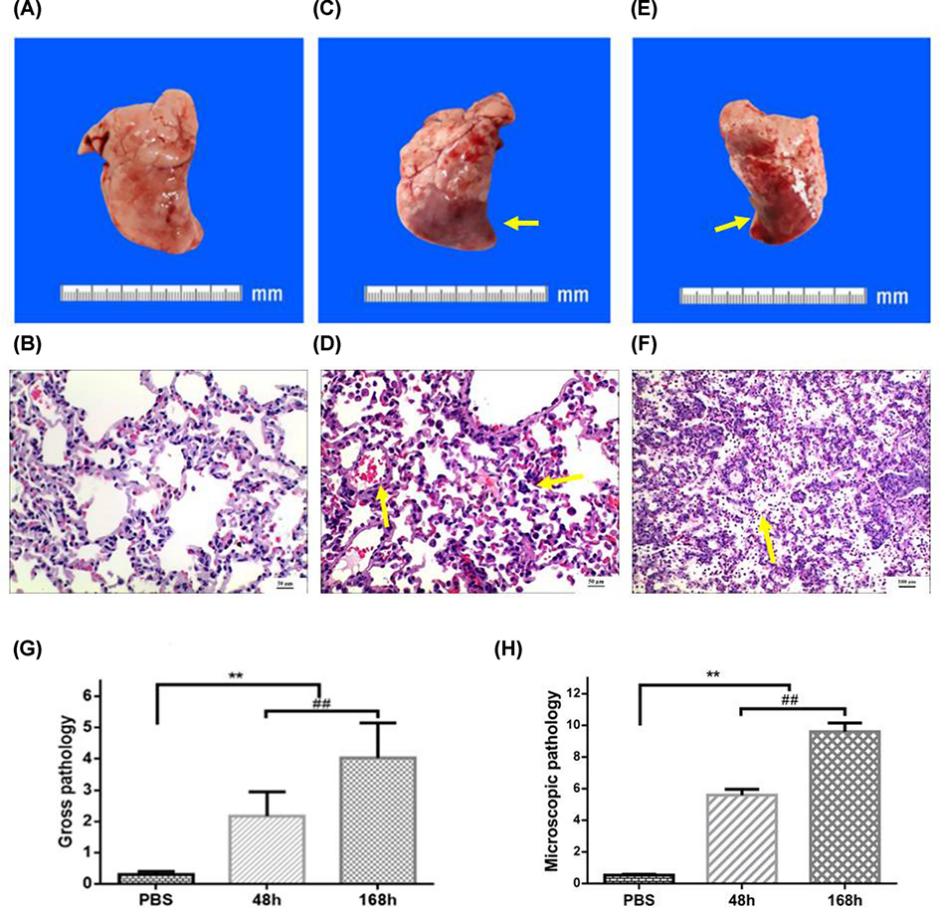
Pulmonary gross and pathology in MRSA-infected rabbit lung (**A**) The lungs of control group had a normal appearance. (**B**) Microscopic examination of the right lower lung from control groups demonstrated normal histology. Bar = 50 μm. (**C**) The lungs of model group at 48 h appeared dark brown, mild hyperemia, and focal consolidation (arrow). (**D**) Microscopic examination of the right lower lung from model groups at 48 h displayed normal lung tissue structure disappearence, angiectasis, congestion (arrow), and many lymphocytes infiltration of alveolar septum (arrow). Bar = 50 μm. (**E**) The lesion of lower right lung of the model group at 168 h was flaky peripheral bleeding, hemorrhagic cut surface of the parenchyma, diffuse consolidation. (**F**) The pulmonary pathology of model group showed suppurative changes at 168 h (arrow). Bar = 100 μm. Results from semiquantitative scoring of gross (**G**) and microscopic pathology (**H**) are shown. **P<0.01 Model group at 48 and 168 h vs control group; ^##^*P<*0.01 Model group at 168 vs 48 h.

Next, we performed visual inspection and manual examination of the lungs excised at necropsy for the assessment of potential differences in control group and model group. Compared with the control group (Figure 3A), lungs from model group had marked changes such as dark brown, focal consolidation, and mild hyperemia at 48 h (Figure 3C). The lesion of lower right lung of the model group at 168 h was significantly increased compared with the former at 48 h, including a little flaky peripheral bleeding, hemorrhagic cut surface of the parenchyma, and diffuse consolidation (Figure 3E). Then we used previously verified criteria semiquantitatively scored each lung to compare the severity of gross pulmonary pathology in a more objective manner. Gross pathology scores for rabbits in the NS sham-infection group ranged from 0.2 to 0.4 (mean score = 0.30). Whereas in the model group, the scores ranged respectively from 1.3 to 2.8 and 3.2 to 4.9 (mean score = 2.17 compared with 4.03) at 48 and 168 h (*P<*0.01, Figure 3G). These gross pathology findings were positively associated with the above clinical examination and bronchoscopy observations.

### Microscopic pathology of the MRSA-infected rabbit lung

To further evaluate MRSA virulence in the lower respiratory tract, we then performed detailed microscopic examination on the lung tissues. Lungs from three NS sham-infected rabbits exhibited no histological evidence of infection (Figure 3B), whereas lungs from all MRSA-infected rabbits had a range of microscopic features of significant lower respiratory tract infection at 48 and 168 h (Figure 3D,F). Three MRSA-infected animals had relatively mild bronchopneumonia at 48 h, such as normal lung tissue structure disappearance, consolidation state, a large number of lymphocytes and eosinophil infiltration, bronchial submucosal and alveolar septal vascular congestion and edema, vasodilation congestion, extensive bleeding, alveolar septum many lymphocytes (Figure 3D), the other three MRSA-infected animals had severe infections at 168 h, showing suppurative changes in the lesion tissue and distributing a large number of pus, neutrophils, lymphocytes, and a small amount of eosinophils in the lesion tissue (Figure 3F).

Next, each lung was semiquantitatively scored by two blinded pathologists using previously validated criteria [15]. The mean microscopic pathology scores for the model group were 5.60 and 9.60 at 48 and 168 h, which were dramatically higher than that in the NS sham-infection group (0.58) (*P<*0.01, Figure 3H). There were statistically significant histopathological changes between the microscopic pathology scores at 48 and 168 h (*P<*0.01).

### Host immune response in MRSA-infected rabbits

**Figure 4:**
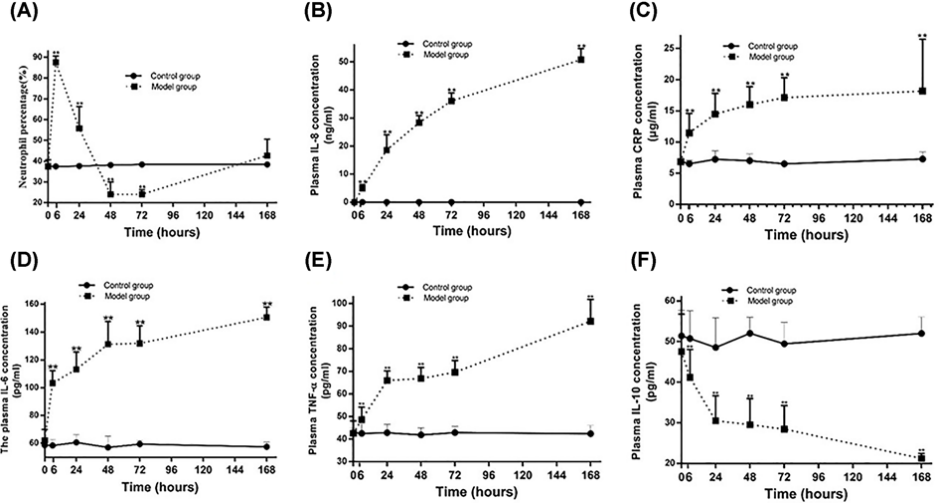
Changes of neutrophil percentage and cytokines in MRSA-infected rabbits (**A**) The change of neutrophil percentage. (**B**) The change of IL-8 in the model group. (**C**) The change of CRP in the model group. (**D**) The change of IL-6 in the model group. (**E**) The change of TNF-α in the model group. (**F**) The change of plasma IL-10 in the model group. **P<0.01 model group vs control group.

Then the percentage of neutrophil and the concentration of cytokines in the peripheral blood and plasma specimens were examined. Neutrophil percentage increased significantly after MRSA infection and reached the peak at 6 h (87.62 ± 3.05 vs 37.52 ± 0.86, *P<*0.01), then decreased gradually. It reached a minimum at 72 h (24.08 ± 2.21 vs 38.45 ± 0.79, *P<*0.01), then slightly increased to a similar level of control group at 168 h (Figure 4A). There was a similar tendency in the change of white blood cells (data not shown). Consistent with a vigorous local and systemic innate immune response, the concentration of CRP, IL-8, IL-6, and TNF-α raised step by step with the prolong of the model time, and remarkably statistical significances were observed at 6, 24, 48, 72, and 168 h compared with the control group (*P<*0.01, Figure 4B–E). But plasma IL-10 concentration was dramatically decreased in model group compared with control group at 6, 24, 48, and 168 h (*P<*0.01, Figure 4F).

Collectively, all the above data demonstrated that a rabbit pneumonia model was successfully constructed by endotracheal directed instillation of 1 × 10^10^ CFU of MRSA using a bronchoscope.

### Clinical assessment of the treatment effect of co-administering hUMSCs and LZD on MRSA-infected rabbit pneumonia

The clinical symptoms, such as frequent cough, nasal yellow secretions, shortness of breath, decreasing food intake, and vitality, were similar with above description at 24 h in all groups. But there was significant improvement in LZD and combined groups than model group from 48 to 168 h (Table 1). Meanwhile, we found some clinical parameters (a frequent cough, wet rales of lung and hemoglobin oxygen saturation) of lower respiratory tract infection in combined group were better than that in LZD group at 168 h (Table 1).

**Figure 5:**
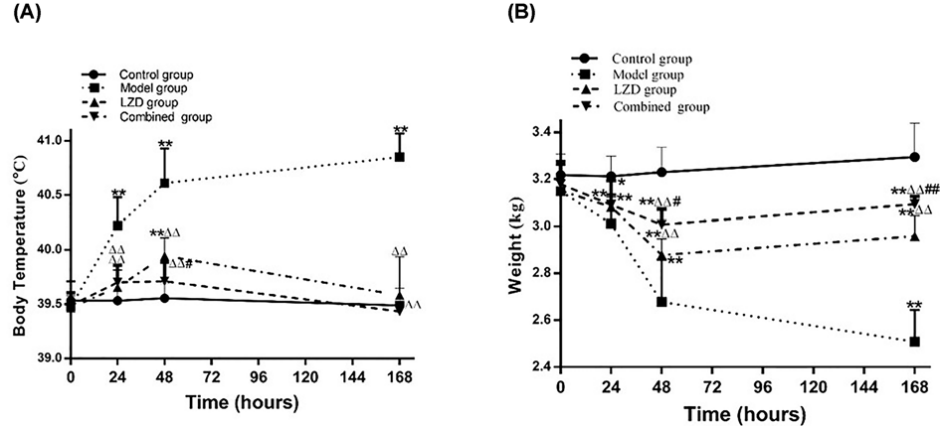
Changes of temperature and weight after MSC combined with Linezolid co-therapy in the MRSA-infection pneumonia (**A**) Temperature change after MRSA inoculation within 168 h. (**B**) Weight change after MRSA inoculation within 168 h. *P<0.05, **P<0.01 vs control group; ^△△^*P<*0.01 vs model group; ^#^*P<*0.05, ^##^*P<*0.01 vs LZD group.

The body temperature of the combined group and LZD group were significant lower than that of the model group at 24, 48, and 168 h (Figure 5A, *P<*0.01), and decreased to the normal level of control group at 168 h. The temperature of the combined group was lower than that of LZD group at 48 h (Figure 5A, *P<*0.05), but there was no significant difference between combined group and LZD group at other times. Except for the continuous decreasing body weight of model group, the body weight of LZD and combined groups decreased gradually and peaked at 48 h, then increased within 168 h. The recovery of body weight of combined group was faster than that of LZD group, and significant difference was observed between groups at 168 h (*P<*0.01, Table 1 & Figure 5B).

**Figure 6:**
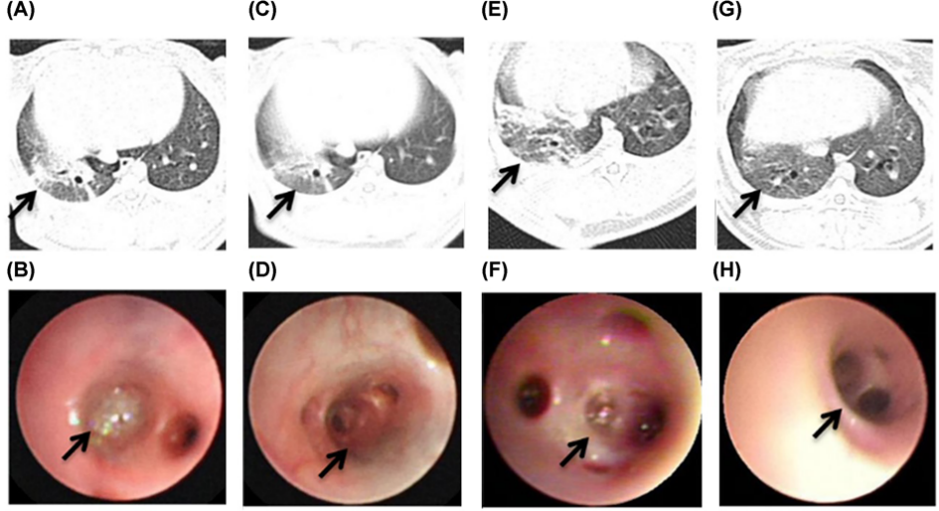
The performance of CT and bronchoscopy after co-therapy of hUMSCs combined with LZD in the MRSA-infection rabbit pneumonia The area of pulmonary consolidation of LZD group (arrow) was observed by CT at 48 (**A**) and 168 h (**C**). The purulent secretion congestion (arrow) could be seen in the bronchus of right lower lobe under the bronchoscope at LZD group at 48 (**B**) and 168 h (**D**). The pulmonary consolidation (arrow) of combined group was observed by CT at 48 (**E**) and 168 h (**G**). The mucosal hyperemia and erosion (arrow) of combined group of LZD group at 48 (**F**) and 168 h (**H**) was observed under the bronchoscope.

Next, the performance of CT and bronchoscopy in combined, LZD and model group were compared at 48 and 168 h. CT showed that the area of pulmonary consolidation of combined and LZD groups were smaller than that of model group at 48 h (Figures 6A,E & 2C). The mucosal hyperemia and erosion of combined group alleviated slightly compared with LZD group at 48 h (Figure 6B,F). Furthermore, we found that the pulmonary consolidation of right lower lobe was shrink in LZD group at 168 h (Figure 6C), and was absorbed basic completely with a small amount of fiber cords left in combined group (Figure 6G). The secretion of tube cavity decreased significantly in both combined and LZD groups. While the mucosal hyperemia and erosion of combined group reduced significantly compared with LZD group under the bronchoscope at 168 h (Figure 6D,H). Simultaneously, the improvement of the distal bronchial ventilation was observed (Table 1).

### The effect of co-administering hUMSCs and LZD on the gross and microscopic pathology of MRSA-infected rabbit lung

**Figure 7:**
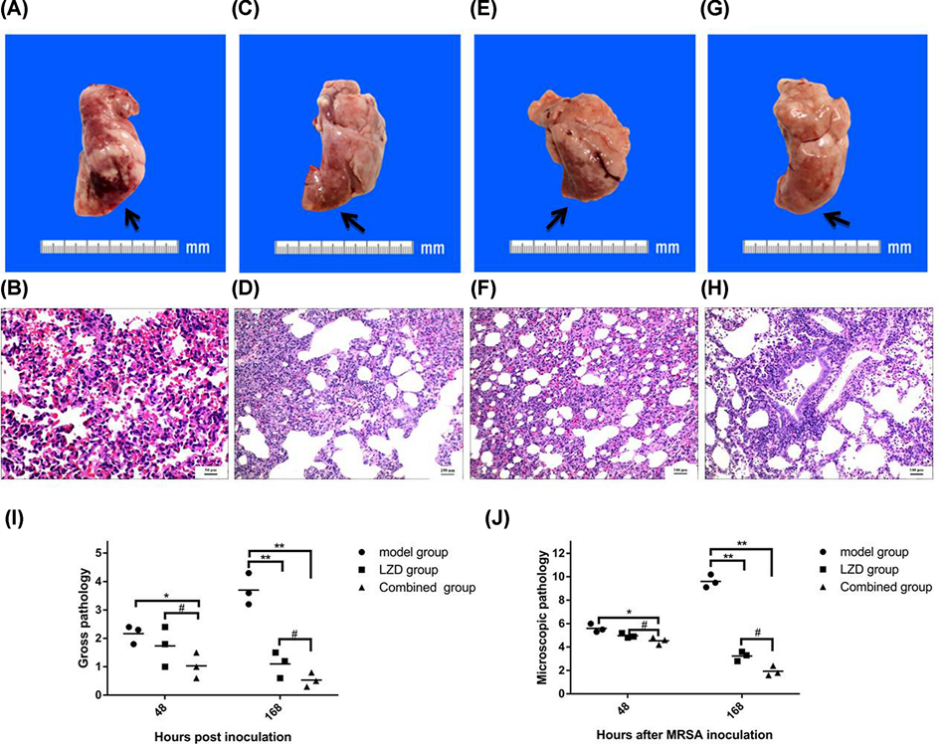
The effect of co-administering hUMSCs and LZD on the gross and microscopic pathology of MRSA-infected rabbit lung (**A**) The lower right lung of LZD group was still dark brown and had patchy hemorrhage at 48 h. (**B**) The pathological changes included the disappeared the lung tissue structure, mononuclear cells in the alveolar cavity, and few lymphocytes infiltration in the tissue interva in LZD group. Bar = 50 μm. (**C**) The color of the lower right lung of LZD group at 168 h was thin and the lesion was smaller than that at 48 h. (**D**) Pathological changes have the alveolar interval fracture, pulmonary interval vasodilation and congestion, and infiltration of many lymphocytes. Bar = 100 μm. (**E**) The color of the lower right lung of combined group was lighter than that of LZD group at 48 h. (**F**) Pathological changes in combined group reduced slightly than that of LZD group at 48 h. Bar = 100 μm. (**G**) The lung consolidation area was significantly reduced and the color restored basically to normal at 168 h. (**H**) The inflammatory exudation at 168 h decreased significantly than that at 48 h. Bar = 100 μm. Results from semiquantitative scoring of gross (**I**) and microscopic pathology (**J**) are shown. *P<0.05, **P<0.01 LZD and combined group at 48 and 168 h vs model group; ^#^*P*<0.05 LZD group vs combined group at 48 and 168 h.

The lower right lung of LZD group was still dark brown and had patchy hemorrhage at 48 h (Figure 7A). Basic pathological changes [15] included: disappeared lung tissue structure, mononuclear cells in the alveolar cavity, a few lymphocytes infiltration in the tissue interval, hyperemia and hemorrhage of expansional pulmonary vascular, many lymphocytes and the eosinophil infiltration around the bronchi and small arteries in LZD group (Figure 7B). At 168 h, the color of the lower right lung of LZD group was thin and the lesion was smaller than that at 48 h (Figure 7C). Pathological changes includes: the alveolar interval fracture, pulmonary interval vasodilation and congestion, infiltration of many lymphocytes, and neutrophils and eosinophils [15]. In addition, inflammatory exudation was significantly reduced compared with model group (Figures 7D & 3E). At 48 h, the color of the lower right lung of combined group was lighter and the lesion size reduced slightly than that of LZD group (Figure 7E). Pathological changes in combined group reduced slightly compared with LZD group (Figure 7F). Compared with that at 48 h, the lung consolidation area in combined group was significantly reduced and the color restored basically to normal at 168 h (Figure 7G). Although there were lymphocytes, neutrophils, eosinophils infiltration in the lung interval, the inflammatory exudation in combined group at 168 h decreased significantly than that at 48 h (Figure 7H). Gross pathology scores for rabbits ranged from 1.0 to 2.4 and 0.6 to 1.5 (mean score is 1.89 compared with 1.48) in the LZD group, and 0.4 to 1.4 and 0.3 to 0.8 (mean score is 0.92 compared with 0.43) in the combined group at 48 and 168 h (*P<*0.01, Figure 7I). The mean microscopic pathology score for rabbits respectively were 4.97 and 3.23 in the LZD group and 4.53 and 1.93 in the combined group at 48 and 168 h (Figure 7J). There were statistically significant histo-pathological changes by semiquantitative scoring in the combined group and LZD group at 48 and 168 h (*P<*0.01). No single microscopic feature disproportionately contributed in this difference in score.

### The effect of co-administering hUMSCs and LZD on host immune response of MRSA-infected rabbits

**Figure 8:**
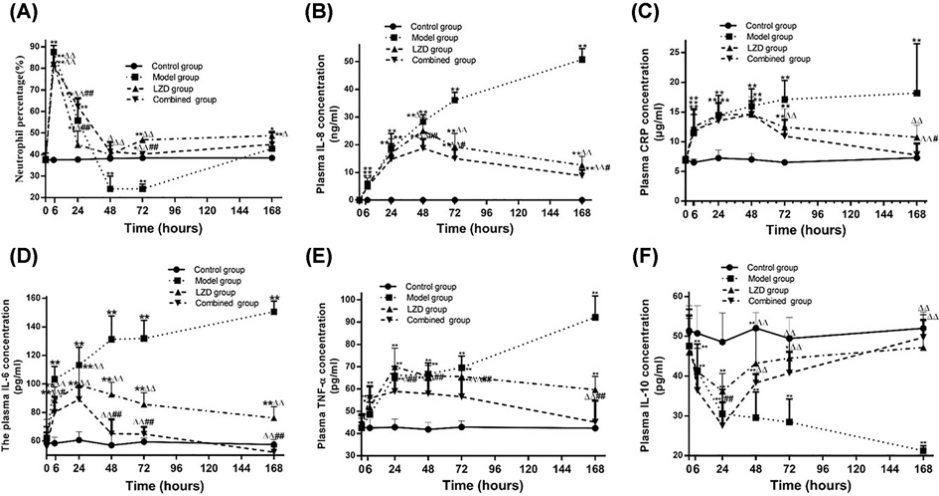
Change of neutrophil percentage and cytokines in MRSA-infected rabbits co-treated with hUMSCs and LZD (**A**) The changes of neutrophil percentage. (**B**) The changes of plasma IL-8 concentration. (**C**) The changes of plasma CRP concentration. (**D**) The changes of plasma IL-6 concentration. (**E**) The changes of plasma TNF-α concentration. (**F**) The changes of plasma IL-10 concentration. *P<0.05, **P<0.01 vs control group; ^△^*P*<0.05, ^△△^*P*<0.01 vs model group; ^#^*P*<0.05, ^##^*P*<0.01 vs LZD group.

The percentage of neutrophil and the concentration of cytokines were measured to assess the therapeutic effect of hUMSCs combined with LZD on MRSA induced lower respiratory tract infection. Neutrophil percentage increased significantly in model group, LZD and combined group compared with the control group, and reached the peak at 6 h (*P<*0.01), but there was no obvious statistical significance among these three groups. Then they decreased gradually and reached the low peaks at 72 h. The low peaks of combined and LZD groups were higher than that of model group (*P<*0.01, Figure 8A). Then they were slightly increased and no obvious statistical significance was observed between them at 168 h (Figure 8A). Consistent with a vigorous local and systemic innate immune response, plasma CRP and IL-8 concentrations were significantly elevated in LZD and combined group within 48 h, then they were gradually decreased. Moreover, the concentrations were significantly lower in combined group than that in LZD group at 168h (*P<*0.05) and a remarkable decrease was observed in combined and LZD group compared with model group at 72 and 168 h (*P<*0.01, Figure 8B,C). The plasma IL-6 and TNF-α concentrations were significantly elevated in LZD and combined group within 24 h, then gradually decreased. The plasma IL-6 concentration of combined group was significantly lower than LZD group at 72 and 168 h (*P<*0.01, Figure 8D) and the plasma TNF-α concentration of combined group was remarkably lower than LZD group at 48, 72, and 168 h (*P<*0.01) (Figure 8E). The plasma IL-10 concentration was gradually decreased in model group within 168 h. However, the plasma concentration of IL-10 reached the low peak at 24 h and then increased gradually in combined and LZD groups. The concentration of IL-10 was significantly lower in combined group than that in LZD group at 24 h (*P<*0.01). There was no statistical significance between combined and LZD group at 48, 72, and 168 h. The plasma IL-10 concentration of combined and LZD groups were comparable with the control group and noticeably higher than that of model group at 168 h (*P<*0.01) (Figure 8F).

## Discussion

In the present study, we investigated the treatment effect of co-administration of hUMSCs and LZD on MRSA-induced pneumonia in a rabbit model. At first, we established the rabbit pneumonia model, which was different from the previous experiments that focussed on disperse intratracheal administration in rodents [17–19]. Although different MRSA-infected animal models have been described previously [16,17,19], there were rarely animal models using implantation of MRSA quantitatively and directionally to special bronchus which mimic the course of lung infection occurred in humans.

The pneumonia model was established by following the methods of previously described with minor modifications [20], where we innovatively used child superfine bronchoscope to inject MRSA directionally and quantitatively into the rabbit basal segment of lower lobe of right lung via nose cavity, which solved the problem of diffuse lung inflammation caused by bacteria injection in the air tube in the previous pneumonia model of small animals [17–19]. Our results showed the rabbits received 1 × 10^10^ CFU of MRSA presented severe clinical and intrapulmonary infection features at 48 and 168 h, respectively, including hypoxemia, lung necrosis, alveolar hemorrhage, pulmonary edema, infiltration of inflammatory cells, and even death. These features mimic largely the course of severe MRSA pneumonia which was observed in humans [21,22].

Undoubtedly immune system plays key roles in the MRSA-induced severe pneumonia [23,24]. *S. aureus* activates multiple excessive proinflammatory signaling cascades, which cause multiorgan failure, collapse of the circulatory system, and even death [23,26]. So, most patients died from imbalance between pathogen and immune system [5,25,26]. Speculatively combined protocol such as using antibiotics to target on pathogens, and meanwhile drugs to limit inflammation reaction should result in more effective treatment outcomes. U.K. treatment guidelines recommend use of linezolid for the treatment of patients with severe staphylococcal necrotizing pneumonia [27]. Our results showed that early treatment with Linezolid improved survival outcomes, reduced lung inflammation and decreased secretion of inflammatory cytokines, such as IL-8, consistent with literature [28]. MSCs is one type of adult stem cells endowed with various biological function such as modulation of inflammation and tissue injury healing. Consistently protective effects of MSCs on bacterial pneumonia have been reported [29–32]. As expected, our results demonstrated that co-administration of hUMSCs and LZD was more effectively controlled MRSA-induced severe pneumonia than LZD alone in terms of improvement in clinical performance, gross, microscopic pathology, and the inflammation. These data suggested that combinational therapy of hUMSCs and LZD might be effective in the treatment of MRSA-induced pneumonia in human.

Difference in the route of MSC administration may affect treatment efficacy of MSCs. Previous studies showed that intratracheal administration of MSC increased alveolar IL-10, KGF and LL-37 expression, and did not reduce bacterial load and lung injury [33,34]. Based on these findings, we chose intravenous administration of hMSCs in our study.

In our previous clinical study, infusion of hUMSCs (1 × 10^6^/kg) significantly alleviated disease activity and clinical symptoms of a subgroup of the patients with Type 2 diabetes mellitus (T2DM) [13]. Refer to this treatment protocol, we applied 1×10^6^/kg as the dose of hUMSCs in the current study. Moreover, this dose was similar to the one used in another study on MRSA pneumonia in rats [29].

It has been reported by Mei et al. that the cytokine storm explored after 6–12 h when inflammation occurred, and MSC administration may be less effective when exceeding the window [35]. In our experiment, we found CRP and neutrophile granulocytes, which were early events in inflammation, amounted to the peak at 6 h. Thereby, taking Mei’s findings together, we proposed 6 h was the beneficial time for MSCs administration. Our present results illustrated that co-administration of MSC and LZD significantly decreased the plasma concentrations of systemic cytokines such as CRP, IL-8, IL-6, and TNF-α in MRSA-induced pneumonia compared with LZD treatment alone, which were in line with previous report [35]. However, different from their results, level of IL-10 showed no difference between LZD and combined groups. These differences may be explained by variations in animal models. Moreover, we found a significant increase in the percentage of neutrophils at 6 h after MRSA injection, then declined gradually. The tendency of neutrophils changes in MRSA-induced pneumonia was consistent to previous reports [36]. However, our study showed that the peak time of neutrophils elevation, 6 h after MRSA injection, was significantly earlier than that of Feng et al. at 24 h. This may be related to that more neutrophils were released by bone marrow in the beginning of MRSA stimulation, then decrease in neutrophil may be associated with Phenol-soluble modulins (PSMs)-induced neutrophil necroptosis [19]. Collectively, these data suggested that hUMSCs treatment possibly reduced pneumonia symptoms via suppression of inflammation induced by MRSA.

In conclusion, our study illustrated that co-administration of hUMSCs and LZD increased survival, reduced lung injury, and enhanced bacterial clearance when used for bacterial pneumonia treatment in rabbits. These findings highly suggest a clinical potential of MSCs in the treatment of severe lung infection.

## Abbreviations

ALI: acute lung injury
ATCC: American Type Culture Collection
CFU: colony forming unit
CRP: C-reactive protein
hUMSC: human umbilical cord-derived MSC
IL: Interleukin
LZD: linezolid
MRSA: methicillin-resistant *Staphylococcus aureus*
NP: nosocomial pneumonia
NS: normal saline
PCT: procalcitonin
PSM: phenol-soluble modulin
TNF: tumor necrosis factor
